# Antioxidant Thymoquinone and Its Potential in the Treatment of Neurological Diseases

**DOI:** 10.3390/antiox12020433

**Published:** 2023-02-09

**Authors:** Nickolay K. Isaev, Elizaveta E. Genrikhs, Elena V. Stelmashook

**Affiliations:** 1 Research Center of Neurology, 125367 Moscow, Russia; 2 Biological Faculty, M.V. Lomonosov Moscow State University, 119991 Moscow, Russia

**Keywords:** thymoquinone, ischemia, Alzheimer’s disease, Parkinson’s disease, traumatic brain injury, oxidative stress, neuroprotection

## Abstract

Oxidative stress is one of the main pathogenic factors of neuron damage in neurodegenerative processes; this makes it an important therapeutic target to which the action of neuroprotectors should be directed. One of these drugs is thymoquinone. According to modern data, this substance has a wide range of pharmacological activity, including neuroprotective, which was demonstrated in experimental modeling of various neurodegenerative diseases and pathological conditions of the brain. The neuroprotective effect of thymoquinone is largely due to its antioxidant ability. Currently available data show that thymoquinone is an effective means to reduce the negative consequences of acute and chronic forms of cerebral pathology, leading to the normalization of the content of antioxidant enzymes and preventing an increase in the level of lipid peroxidation products. Antioxidant properties make this substance a promising basis for the development of prototypes of therapeutic agents aimed at the treatment of a number of degenerative diseases of the central nervous system.

## 1. Introduction

Oxidative stress is one of the main pathogenic factors that cause the death and damage of nerve cells in a number of neurodegenerative diseases and pathological conditions of the nervous system. In the body, oxidative stress is caused by an excess of free radicals, which damage all components of cells, including DNA, RNA, proteins, and lipids. Oxidative degradation of lipids leads to the formation of malondialdehyde (MDA) and 4-hydroxynonenal, as well as isoprostane from unsaturated fatty acids. Protein damage can occur during thiol oxidation, carbonylation, and side chain oxidation, fragmentation, unfolding, and misfolding, leading to loss of activity. Additionally, 8-hydroxydeoxyguanosine is an indicator of DNA damage [1]. The greatest danger of oxidative stress is associated with the formation of reactive oxygen species (ROS), which include hydroxyl radical (HO^•^), superoxide anion (${O_{2}}^{•-}$), hydrogen peroxide (H_2_O_2_), nitric oxide (NO^•^), and peroxynitrite (ONOO^−^). In addition, ions of metals such as iron and copper can participate in the redox cycle, catalyzing the formation of free radicals and ROS. Under normal conditions, the intracellular content of these active molecules is maintained at a low level by various enzyme systems involved in redox homeostasis. Thus, oxidative stress can develop both as a result of hyperproduction of free radicals in the body, and as a result of a decrease in the activity of antioxidant systems. One of the main generators of ROS in the cell is peroxisomes, in which the enzymes forming hydrogen peroxide are localized. Normally, all peroxide formed in peroxisomes is utilized inside these organelles. In addition, in the smooth endoplasmic reticulum, a number of cytochrome-dependent oxygenases producing superoxide ${O_{2}}^{•-}$ are localized. Various enzymatic systems, such as the NADP(H)-oxidase system, cytochrome P450 (a class of proteins responsible for detoxification of organic poisons), and xanthine oxidoreductase are able to produce superoxide anions in the cytoplasm. Significant sources of ROS in cells are mitochondria, whose electron transport chain is a constant source of ROS. Normally, from 1 to 3% of the absorbed oxygen is converted into ROS, most of which are formed due to single electron reduction in complex I (NADH CoQ reductase) and complex III (CoQ cytochrome c reductase). Moreover, various redox-active compounds capable of being restored in the respiratory chain of mitochondria stimulate the formation of ROS. For example, such compounds include paraquat, diquat, benzyl viologen, menadione, doxorubicin, etc.

The brain is very susceptible to oxidative stress due to its high oxygen consumption, rich lipid content, and weak antioxidant capacity [2,3,4]. In the brain, the main protection against oxidative stress is provided by the antioxidant enzymatic system (superoxide dismutase (SOD), glyoxalase, glutathione reductase, glutathione peroxidase and catalase), and low molecular weight antioxidants (glutathione, uric acid, ascorbic acid, and melatonin) [3]. Antioxidant protection of the cell is associated with the redox-sensitive transcription factor, a nuclear factor erythroid 2-related factor 2 (Nrf2), which regulates the expression of antioxidant proteins that protect against oxidative damage [5]. Thus, with normal physiological functioning in cells, a dynamic balance is maintained between the processes of ROS formation and the oppositely directed action of the protective antioxidant system. However, prolonged intensification of ROS production in the brain in ischemia, traumatic brain injury, and other pathological conditions leads to depletion of the antioxidant system, oxidative stress, the development of inflammatory processes, and cell death [6,7]. Currently, there is an intensive search for exogenous antioxidants that can resist the development of oxidative stress during neurodegenerative processes of the brain. One of the promising natural antioxidants of plant origin is thymoquinone (TQ; 2-isopropyl-5-methyl-1,4-benzoquinone); it is found in black cumin seeds (Nigella sativa, Ranunculaceae), where its content ranges from 30 to 48% [8]. TQ was first synthesized in 1910 by the oxidation of thymol (2-isopropyl-5-methylphenol) with hydrogen peroxide [9], and is a bright yellow crystalline compound. Numerous experimental studies performed on in vitro and in vivo models showed anti-inflammatory, antihypertensive [10], antiasthmatic [11], antidiabetic [12], and antitumor [13] effects of TQ. A significant number of scientific groups showed pronounced antioxidant properties of this compound [14].

## 2. Antioxidant Properties of Thymoquinone in Models of Neurodegeneration Associated with Acute Processes of Brain Damage

### 2.1. Antioxidant Properties of Thymoquinone in Brain Ischemia Models

Acute cerebral circulatory disorders (strokes) are the leading cause of disability in the population, as well as the second leading cause of death worldwide [15]. According to a number of data, motor disorders after an acute period of stroke are observed in 85% of patients, and by the end of the first year, they persist in 70%. It should be noted that after a stroke, not only motor disorders occur. Immediately after the acute period of stroke, 36% of patients have speech disorders (aphasia) [16]. The main factors causing the death of neurons after ischemia are oxygen and glucose deprivation, resulting in oxidative stress, acidosis, and glutamate cytotoxicity. Not only the ischemic period is dangerous, but also reperfusion, which leads to the intensive formation of very dangerous ROS, causing oxidative stress, which is responsible for most of the ischemic-reperfusion damage to brain tissue [17]. Currently, in the field of neuronal, endothelial, and glial protective therapy a large number of studies were carried out; however, there is no real therapeutic strategy in ischemic-reperfusion for brain damage yet [18]. Based on the pathophysiology of brain damage, the therapeutic effect in this pathological condition should be aimed primarily at reducing the excess of ROS and inhibiting inflammatory cascades [19]. Undoubtedly, it is necessary to search and study pharmacological drugs with pronounced antioxidant properties. In recent years, such studies focused on TQ, which has potential neuroprotective and antioxidant properties [20]. Using a model of transient cerebral ischemia, it was demonstrated that daily administration of TQ to animals increased the survival of neurons in the hippocampal CA1 field, maintained normal levels of glutathione, SOD and catalase, and also reduced the level of MDA in brain tissue [21] (Table 1). In these experiments, TQ (5 mg/kg) was administered to animals for 5 days before ischemic exposure, as well as during reperfusion. Transient ischemia was caused by 10 min bilateral occlusion of both common carotid arteries followed by reperfusion. Pronounced antioxidant properties of TQ are also shown in the model of global ischemia initiated by 20-minute four-vessel occlusion of the carotid arteries in rats. In this case, 10 mg/kg of TQ was administered to animals immediately before and in the next 2 days after ischemia, which led to a significant decrease in the level of MDA compared to the ischemic group [22]. The reduction in ischemic brain damage under the action of TQ can be largely associated with the activation of the nuclear erythroid 2-related proteins and heme-oxygenase-1 pathway by thymoquinone [23]. In vivo and in vitro experiments showed that activation of the nuclear erythroid 2-related proteins and heme-oxygenase-1 signaling pathway is necessary for the manifestation of such protective effects of TQ as prevention of cell death, reduction in inflammation and oxidative stress, prevention of apoptosis, and autophagy. The authors of this study concluded that TQ is a promising drug whose action can be directed to the therapy of cerebral ischemia; its effect is mediated by a decrease in oxidative stress and neuronal cell death [23]. Interesting data were obtained on a model of global cerebral ischemia caused by permanent bilateral common carotid artery occlusion. In this work, the effect of the water–alcohol extract of *Nigella sativa* seeds and its active component TQ on markers of redox status was investigated. Treatment of animals with both *Nigella sativa* seed extract (400 mg/kg/day) and TQ (40 mg/kg/day) reduced in the hippocampus an increase in the level of lipid peroxidation caused by ischemia and prevented a decrease in the SOD activity [24].

However, TQ is a rather hydrophobic compound, which complicates the administration of this substance to animals. Due to its low solubility and poor absorption, this substance reaches low levels in blood serum and tissues. Therefore, in one of the works, an intranasal mucoadhesive nanoemulsion containing TQ, obtained by ion gelation, was used as a prototype of a pharmacological preparation. In the model of cerebral ischemia/reperfusion in rats, this drug proved to be more effective and had increased bioavailability to brain tissues compared to intravenous administration of TQ [8]. It should also be noted that the intranasal administration of nanoparticles optimized by the poly(lactide-co-glycolide)+chitosan complex and loaded with TQ to animals with the occlusion of the middle cerebral artery significantly reduced the intensity of lipid peroxidation in brain tissue and led to an increase in the level of glutathione, catalase, and SOD. Intranasal delivery of TQ-poly(lactide-co-glycolide)+chitosan nanoparticles resulted in a higher concentration in the brain, but a lower concentration of TQ in plasma compared to intravenous delivery [25]. The data obtained allow for considering the drugs created on the basis of the inclusion of TQ in nanocarriers as a promising prototype of drugs with a pronounced antioxidant effect. In a later work, a drug was demonstrated in which TQ was part of nanocarriers. The nanoparticles of mesoporous silica or nanoparticles of mesoporous silica coated with a lipid bilayer served as a nanocarrier. It was found that nanoparticles of mesoporous silica coated with a lipid bilayer successfully transported TQ to several brain regions. For example, a significant increase in TQ delivery in the thalamus was shown compared to the group receiving free TQ. However, in the cerebral cortex, the content of TQ was significantly lower compared to the free TQ group. The authors concluded that nanoparticles of mesoporous silica coated with a lipid bilayer are convenient nanoplatforms that can be used to accurately transport drugs to certain brain structures [26].

### 2.2. Antioxidant Properties of Thymoquinone in Traumatic Brain Injury Models

Traumatic brain injury leads to complex brain damage and is a significant cause of disability and mortality. Approximately 1.7 million people suffer from traumatic brain injury, which leads to 52,000 deaths annually [27,28,29]. The severity of the consequences of traumatic brain injury depends on many factors and can vary from ultrastructural damage to mechanical destruction of significant areas of the brain. Traumatic brain injury can lead to widespread neurocognitive, neuroendocrine, speech, and mental dysfunctions. Traumatic brain injury causes a complex of neurodestructive biochemical processes in the brain, including an increase in the production of free radicals, nitric oxide, an increase in the level of intracellular calcium, which leads to a cascade of secondary damage, the development of chronic inflammation, synaptic dysfunction, and neurological disorders [29,30].

Antioxidant therapy is a promising area of treatment for patients with traumatic brain injury, as it has a minimal number of side effects and can interrupt the developing cascade of secondary brain damage in the early stages. Previously, the positive effect of antioxidants, such as N-Acetylcysteine, resveratrol, and coenzyme Q10, was shown on various models of traumatic brain injury [31,32,33]. However, data on the antioxidant effect of TQ in traumatic brain injury are currently somewhat contradictory. In a model of open unilateral rat traumatic brain injury, it was shown that the administration of TQ (5 mg/kg) to animals after traumatic exposure prevented the death of neurons in the hippocampus and reduced the increase in MDA level caused by traumatic brain injury [34]. Thus, based on the results of this work, it can be concluded that one of the mechanisms of the protective action of TQ in traumatic brain injury is the reduction in oxidative stress in the brain. However, in a later study, it was found that although low doses of TQ exhibit a neuroprotective effect after severe traumatic brain injury, which was expressed in a decrease in neuron edema and partial preservation of Na^+^/K^+^-ATPase activity, TQ did not affect the levels of glutathione and MDA. The results indicate the absence of the antioxidant effect of the drug. In this work, TQ was injected into animals within three hours after the injury [35].

It should be noted that an increase in the production of mitochondrial ROS after traumatic brain injury is the most important pathogenic mechanism of neurodestruction, since it causes selective peroxidation of mitochondrial cardiolipin [36]. Mitochondria-targeted antioxidants are able to effectively neutralize the oxidation of mitochondrial cardiolipin [37]. Compounds in which TQ is used as the antioxidant part of the molecule have the greatest antioxidant activity among the mitochondria-targeted antioxidants [38]. In this case, antioxidant activity is carried out by direct neutralization of ROS due to oxidation of TQ [39]. Using a model of the focal open unilateral traumatic brain injury of the sensorimotor cortex of rats, it was shown that intraperitoneal administration of the mitochondria-targeted antioxidant SkQT1 or SkQTR1 significantly reduced neurological deficit in animals caused by trauma [40].

### 2.3. Antioxidant Properties of Thymoquinone in Models of Neurotoxicity of Metals

Neurotoxicants are any chemical substances, including metal ions, which disrupt the normal functioning of the central and/or peripheral nervous system. Exposure to metals, such as lead, cadmium, and aluminum, can disrupt the functioning of the brain, especially when these negative effects occur at an early age or during embryonic development [41]. The toxic effect of these metals is largely due to their ability to cause oxidative stress in the body [42,43,44]. One of the neurotoxic metals is aluminum, high concentrations of which are found in foundry dust particles and which is widely represented in the environment [45]. It is currently assumed that exposure to aluminum may be a risk factor for Alzheimer’s disease [46]. It is believed that the toxic effect of aluminum is associated with its pronounced prooxidant activity [47], since this metal forms a complex with a superoxide anion, which exhibits a very high reactivity [48].

In animal experiments, it was shown that the administration of AlCl_3_ (intraperitoneal, 6 weeks, 10 mg/kg) led to impaired coordination of movements, caused anxiety and depression, increased the content of MDA in the brain, and reduced the overall antioxidant capacity. All these violations were eliminated if animals received intraperitoneal TQ (10 mg/kg) daily. Thus, TQ may be a promising treatment for neurotoxicity caused by AlCl_3_ [49]. Lead is even more dangerous for living organisms, especially for pregnant women and young children. In an experimental model of chronic lead intoxication of pregnant animals, it was shown that the administration of lead to rats caused dose-dependent toxicity for both the maternal organism and embryos. In the brains of pregnant animals and embryos treated with lead, a significant increase in the level of MDA and a decrease in the level of activity of such antioxidant enzymes as SOD, catalase, and glutathione peroxidase were found; it was reliably prevented by TQ treatment. Thus, the treatment of TQ with the toxic effect of lead can mitigate the prooxidant effects in the brain of the mother and fetus induced by this heavy metal [50].

Another very toxic metal is arsenic. The World Health Organization estimates that more than 200 million people worldwide are currently chronically exposed to arsenic [51]. Contaminated drinking water is the main route of arsenic entering the animal and human bodies, which leads to the formation of ROS and the initiation of lipid peroxidation in the brain, a decrease in the level of glutathione, and in the activity of antioxidant enzymes glutathione reductase and SOD [52]. In animal experiments, exposure to arsenic in the form of sodium arsenate (10 mg/kg/day; orally) caused neurobehavioral disorders and the development of oxidative stress in the hippocampus accompanied by activation of lipid peroxidation and the formation of protein carbonyls, a decrease in SOD and glutathione levels. Administration of TQ to animals (2.5 and 5 mg/kg per day, orally) three days before the administration of arsenic significantly weakened the arsenic-caused oxidative stress observed in the hippocampus [53]. Neuroprotective and antioxidant effects of TQ when toxic metals act on the brain may be related to the fact that TQ could upregulate nuclear factor (erythroid-derived 2)-like 2 (Nrf2)/glutamate-cysteine ligase catalytic subunit and Nrf2/glutamate-cysteine ligase modifier subunit pathway. Enhanced regulation of Nrf2/ glutamate-cysteine ligase signaling promotes glutathione synthesis and attenuation of oxidative stress [54].

**Table 1:** Antioxidant properties of thymoquinone (TQ) in models of neurodegeneration associated with acute processes of brain damage.

Model	The Order of Administration and Form of the Drug	Antioxidant Effect	Reference
Transient cerebral ischemia (10-min bilateral occlusion of both common carotid arteries) in rats	Daily administration of TQ (5 mg/kg) was administered to animals for 5 days before ischemic exposure, as well as 7 days during reperfusion per os.	Normalizes the levels of glutathione, SOD, catalase, and also reduces the level of MDA in hippocampus.	[21]
Global ischemia initiated by 20-min four-vessel-occlusion of the carotid arteries in rats	10 mg/kg of TQ was administered to animals immediately before and in the next 2 days after ischemia i.p.	Decreases the level of MDA in brain.	[22]
Permanent bilateral common carotid artery occlusion in rats	*Nigella sativa* seed extract (400 mg/kg/day) and TQ (40 mg/kg/day) i.p.	Reduction in lipid peroxidation and prevention of a decrease in the SOD activity.	[24]
Middle cerebral artery occlusion in rats	Intranasal administration of nanoparticles optimized by the poly(lactide-co-glycolide)+chitosan complex and loaded with TQ for 12 days (50 μL in each nostril).	Reduces the intensity of lipid peroxidation in brain tissue and leads to an increase in the level of glutathione, catalase, and SOD.	[25]
Open unilateral rat traumatic brain injury	TQ (5 mg/kg) was administered to animals after traumatic exposure for 7 days i.p.	Reduces the increase in MDA level.	[34]
Intraperitoneal administration of AlCl_3_ (6 weeks, 10 mg/kg)	Intraperitoneal administration of TQ (10 mg/kg) daily.	Reduces the content of MDA in the brain and prevents a decrease in the overall antioxidant capacity.	[49]
Lead treatment of pregnant rats. Lead administration (160 and 320 ppm) started from gestation day 1 to day 20. Per os	Pb and TQ co-administration. Females received TQ (10 mg/kg body weight) per os.	Decreases the content of MDA and prevents a decrease in SOD, catalase, glutathione peroxidase level in the fetal, and females brain.	[50]
Arsenic in the form of sodium arsenate (10 mg/kg/day; orally) to rats	TQ (2.5 and 5 mg/kg per day, orally) three days before the administration of arsenic.	Decrease in protein carbonyl and lipid peroxidation, prevention of decrease in level of glutathione and SOD.	[53]

## 3. Chronic Neurodegenerative Diseases

### 3.1. Neuroprotective Properties of Thymoquinone in Modeling Alzheimer’s Disease

Alzheimer’s disease is a neurodegenerative disease, the prevalence of which is increasing every year. Currently, there are about 50 million patients worldwide with the main clinical manifestations characteristic of this disease: cognitive dysfunction, memory loss, and pathological personality changes. According to the Alzheimer’s Association, this disease accounts for 60–80% of dementia cases [55]. The etiology of the disease and the mechanisms of its development were not sufficiently studied. It is assumed that the main triggers of neurodegenerative processes in Alzheimer’s disease are beta-amyloid peptide and hyperphosphorylated intracellular tau protein. The predominance of the rate of formation of beta-amyloid in the brain of a sick person over the rate of destruction of this peptide is one of the main reasons for the progression of Alzheimer’s disease [56]. Oxidative stress plays a significant role in the pathogenesis of Alzheimer’s disease. An increase in the production of free radicals, a decrease in the activity or expression of antioxidant enzymes, including SOD and catalase, were described both in the central nervous system and in peripheral tissues of patients with Alzheimer’s disease [57,58,59]. The high antioxidant activity and anti-inflammatory properties of thymoquinone created prerequisites for its evaluation as a possible neuroprotector in the modeling of Alzheimer’s disease. According to the literature analysis using the collaborative approach to meta-analysis and review of animal data from experimental studies (CAMARADES) system, a significant therapeutic potential of TQ was shown to be associated with its antioxidant and anti-inflammatory properties [60]. In the work of Kantar et al., the antioxidant properties of TQ were also shown in a scopolamine-induced animal model of Alzheimer’s disease. Scopolamine (1 mg/kg), dissolved in 0.9% saline solution, was administered intraperitoneally (or intravenously) to rats. TQ (20 mg/kg), created in corn oil, was administered intraperitoneally 1 h before the experiments. The authors showed that TQ reduced lipid peroxidation in the brain of animals treated with scopolamine, and concluded that it can be used as an auxiliary therapeutic strategy for the treatment of Alzheimer’s disease [61] (Table 2). We indicated above that exposure to aluminum may be a risk factor for developing Alzheimer’s disease [46], so in some studies, aluminum chloride is used to simulate Alzheimer’s disease in animals [62]. Using such a model, it was shown that AlCl_3_ caused a violation of spatial learning and memory in rats; in their brain tissues, there was a deterioration in total antioxidant capacity, an increase in MDA content, a decrease in the SOD level, and in the expression of Nrf2. Treatment of animals with TQ restored antioxidant activity and increased the levels of Nrf2 in brain tissues [63].

The administration of beta-amyloid peptide into the culture medium of neurons and neuronal cell lines serves as another model of Alzheimer’s disease. Using cultured lines of undifferentiated pheochromocytoma cells (PC 12) and human neuroblastoma cells (SH–SY5Y), it was demonstrated that TQ is able to reduce the cytotoxicity of beta-amyloid, reducing oxidative stress and normalizing mitochondrial function by maintaining physiological levels of matrix metalloproteinases, ROS, and glutathione [64,65]. Similar data were obtained on human cholinergic neurons derived from induced pluripotent stem cells. According to the authors of this work, TQ prevented a decrease in the glutathione levels and an increase in the formation of ROS caused in neurons by the toxic effect of beta-amyloid 1–42 [66].

It should also be noted that beta-amyloid, penetrating into the mitochondria, is able to form oligomers inside them, leading to increased production of ROS by these organelles [67]. ROS cause a violation of long-term synaptic plasticity in the hippocampus, which is the main factor of loss of memory and other cognitive functions associated with Alzheimer’s disease [68,69]. However, a single intraperitoneal administration of a mitochondria-targeted antioxidant, in which TQ was used as the antioxidant part of the molecule, prevented the beta-amyloid-induced inhibition of long-term synaptic plasticity in the hippocampal sections of rats [40].

### 3.2. Neuroprotective Properties of Thymoquinone in Modeling Epilepsy

Epilepsy is one of the most heterogeneous neurological disorders, which is characterized by persistent neural activity and recurrent spontaneous seizures. This neurological disorder can occur for a number of reasons, including as a result of traumatic brain injury and genetic factors [70,71]. Violation of the inhibitory–excitatory balance in the brain is considered as an important mechanism of the pathogenesis of epilepsy. Clinical and experimental data indicate that prolonged and recurrent epileptic seizures can lead to chronic brain damage and neuronal death. Oxidative stress plays a critical role in various forms of neuronal death caused by epileptic seizures [72]. Pentylenetetrazole kindling is a well-established animal model that simulates clinical epilepsy. The essence of the kindling effect is that frequent pre-threshold stimulation increases convulsive readiness and can lead to spontaneous seizures in an experimental animal. In the work of Abdel-Zaher et al., the administration of pentylenetetrazole (35 mg/kg i.p.) to mice once every other day for 12 injections caused kindling accompanied by an increase in MDA, a decrease in the intracellular glutathione levels, and glutathione peroxidase activity in the brain [73]. However, in the case of animal treatment with TQ (5, 10, and 20 mg/kg intraperitoneally, together with a subconvulsive dose of pentylenetetrazole every other day), it caused dose-dependent protection against oxidative stress and from pentylenetetrazole-induced kindling. An earlier study used the temporal lobe epilepsy model, which is based on intrahippocampal administration of kainate to animals. After kainate injection, convulsive activity was observed, the levels of MDA, nitrites, and nitrates were increased, and the activity of superoxide dismutase was decreased. Pretreatment of animals with TQ at a dose of 10 mg/kg orally weakened convulsive activity and reduced lipid peroxidation and loss of hippocampal neurons [74]. Interesting data were obtained on the lithium-pilocarpine rat model of status epilepticus. In this study, rats were intraperitoneally injected with 1% LiCl (3 mg/kg) followed by 1% pilocarpine (30 mg/kg) 20 h later. Treatment of animals with TQ reduced the severity of seizures and increased the expression of Nrf2 proteins, heme oxygenase 1, and SOD in rat hippocampus. The authors suggested that TQ attenuated brain injury induced by status epilepticus via an antioxidant pathway [75].

### 3.3. Neuroprotective Properties of Thymoquinone in Modeling Parkinson’s Disease

Parkinson’s disease is an incurable neurodegenerative disease accompanied by progressive death of dopaminergic neurons, primarily in the substantia nigra. Symptoms of this disease (tremor, hypokinesia, muscle rigidity, and postural instability) manifest themselves with the death of 60–80% of neurons of the substantia nigra [76]. Currently, the development of Parkinson’s disease is associated with the aggregation of α-synuclein, the development of neuroinflammation, and oxidative stress [77,78]. The drugs used in Parkinson’s disease therapy lead only to the mitigation of the symptoms of the disease without prevention of its progression. One of the Parkinson’s disease models is animals injected with 6-hydroxydophamine into the striatum, which leads to a decrease in the number of neurons in the substantia nigra, an increase in the level of MDA, and a decrease in the SOD activity in the brain. Pretreatment of animals with TQ (5 or 10 mg/kg three times at an interval of 24 h) not only prevented the loss of neurons in the substantia nigra, but also prevented an increase in MDA levels [79].

An interesting model of neurodegenerative processes in Parkinson’s disease is the administration of 1-methyl-4-phenyl 1,2,3,6 tetrahydropyridine (MPTP) to animals. MPTP itself is not toxic, and as a lipophilic compound, can cross the blood–brain barrier. Inside the brain, MPTP is metabolized into the toxic cation 1-methyl-4-phenylpyridinium (MPP^+^). MPP^+^ inhibits complex I of the mitochondrial electron transfer chain, which causes increased production of free radicals, the development of oxidative stress, and cell death. In the work of Ardah et al., it was shown that the administration of MPTP to mice caused oxidative stress associated with a decrease in SOD and catalase activity, depletion of reduced glutathione, and an increase in MDA level [80]. However, if TQ (10 mg/kg body weight) was administered to animals 1 week before MPTP, then such treatment prevented a decrease in the activity of antioxidant enzymes, depletion of glutathione, and inhibited lipid peroxidation. It should be noted that TQ not only prevented the toxicity caused by MPTP, but also inhibited the formation of α-synuclein fibrils and related toxicity. In the same model, it was shown that TQ increases the nuclear translocation of Nrf2, the expression of a number of antioxidant genes, such as heme oxygenase 1, quinone oxidoreductase 1, and glutathione-S-transferase. Suppression of Nrf2 by siRNA prevented the development of the protective effects of TQ [81]. In this study TQ was administered to animals intraperitoneally at a dose of 10 mg/kg body weight for 1 week, starting on the day before each dose of MPTP.

**Table 2:** Antioxidant properties of thymoquinone (TQ) in models of chronic neurodegenerative diseases.

Model	The Order of Administration and Form of the Drug	Antioxidant Effect	Reference
Scopolamine-induced model of Alzheimer’s disease in rats	TQ (20 mg/kg, in corn oil) was administered intraperitoneally 1 h before the experiments.	Reduction in lipid peroxidation in the brain.	[61]
AlCl_3_-induced model of Alzheimer’s disease in rats	10 mg/kg of TQ every day by mouth i.p.	Increases the levels of Nrf2 in brain tissues, recovery of total antioxidant capacity.	[63]
Model of Alzheimer’s disease in vitro (administration of beta-amyloid peptide into the culture medium of neurons, cell lines PC 12 and SH–SY5Y)	Medium containing TQ (100 nM) for a period up to 24–48 h.	Reducing oxidative stress, ROS production, and recovery of glutathione level.	[64,65,66]
Pentylenetetrazole kindling-animal model of clinical epilepsy	TQ (20 mg/kg intraperitoneally, together with a subconvulsive dose of pentylenetetrazole every other day).	Decrease in malondialdehyde and recovery of glutathione peroxidase activity, and reduction the glutathione level in brain.	[73]
The temporal lobe epilepsy model (intrahippocampal administration of kainate to animals)	TQ at a dose of 10 mg/kg per os starting 1 week before surgery. The last treatment was carried out 1 hbefore the surgery.	Decrease in malondialdehyde and recovery of SOD activity in hippocampus.	[74]
Lithium-pilocarpine rat model of Status epilepticus	TQ 10 mg/kg twice by intraperitoneal injection 24 h and 1 h prior to injection of pilocarpine.	Increase in Nrf2 and SOD activity in cortex and hippocampus.	[75]
The Parkinson’s disease models (animals injected with 6-hydroxydophamine into the striatum)	Pretreatment of animals with TQ (5 or 10 mg/kg) three times with an interval of 24 h per os.	Prevention of an increase in the MDA levels.	[79]
The Parkinson’s disease models (animals injected with MPTP to mice)	TQ (once daily for 1 week, 60 min prior to each dose of MPTP administration). i.p.	Decrease in malondialdehyde and recovery of SOD, catalase activity, and glutathione level in brain.	[80]

## 4. Further Directions of Research: Limitations of Use of Thymoquinone in Clinical Trials

**Figure 1:**
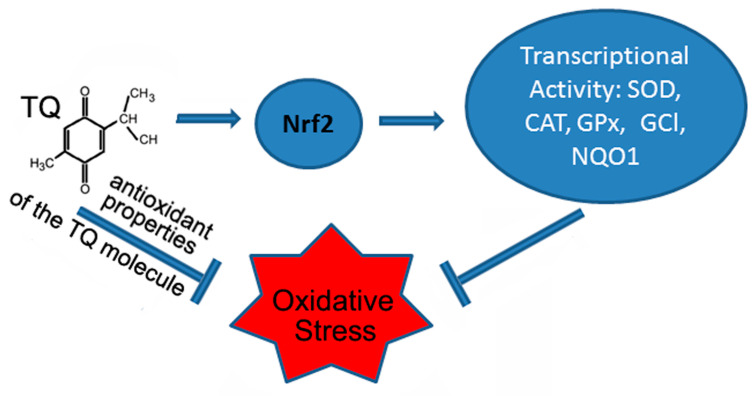
The protective effects of thymoquinone (TQ) under oxidative stress are mediated by activation of the Nrf2 pathway and antioxidant properties of the TQ molecule. Abbreviations in the figure: nuclear factor erythroid 2-related factor 2 (Nrf2), superoxide dismutase (SOD), catalase (CAT), glutathione peroxidase (GPx), glutamate-cysteine ligase (GCL), and NADPH quinone oxidoreductase 1 (NQO1).

Despite the fact that numerous studies reported on a variety of pharmacological and medicinal properties of TQ, clinical trials of TQ were not initiated due to its poor bioavailability. Therefore, currently, attempts are being made to use nanostructured carriers loaded with TQ to eliminate problems with bioavailability [8,26,82]. According to these studies, such drugs proved to be more effective, and increased bioavailability in the brain compared to intravenous administration of TQ [8]. In another study, a suspended self-nanoemulsifying drug delivery system using the microemulsification technique was used to solve these problems; this made it possible to increase the bioavailability of the TQ- self-nanoemulsifying drug delivery system fourfold compared to pure TQ [83]. Another direction of improving the dosage form of the drug is the creation of mitochondria-targeting antioxidants, in which TQ is used as the antioxidant part of the molecule [39,40]. The transport of the drug into the mitochondria is carried out by the transport part of the molecule, which includes the penetrating cation tetraphenylphosphonium or rhodamine 19. This form of the drug freely penetrates into the mitochondria and accumulates there under the influence of the electric field of the inner mitochondrial membrane. Another issue that requires additional research is the mechanism of inhibition of oxidative stress by thymoquinone. It was found that the protective effects of TQ under oxidative stress are mediated to a large extent by stimulation of the expression of the Nrf2 gene and induction of nuclear translocation of Nrf2 (Figure 1).

**Figure 2:**
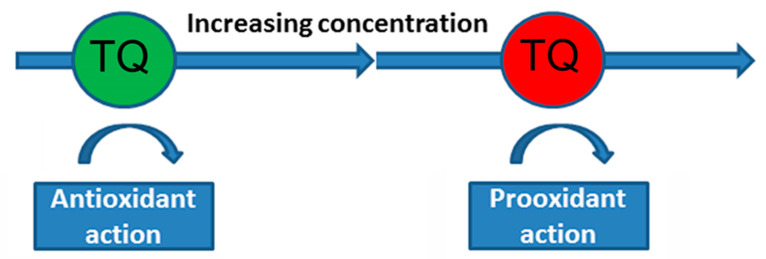
Oxidant/antioxidant effects of thymoquinone (TQ) depend on its concentration.

Nrf2 is a transcriptional factor depending on the basic leucine zipper protein group and codes via NFE2L2 gene. This transcriptional factor performs an important function in regulating the expression of various mammalian antioxidant genes. Activation of Nrf2 can be caused by various stressful effects, including moderate oxidative stress [84]. The modulating effect of TQ on Nrf2 signaling pathways leads to an increase in the activity of the antioxidant systems of the cell (SOD, catalase, glutathione, glutathione peroxidase, and glutathione-S-transferase), to a decrease in lipid peroxidation and, as a result, to a decrease in oxidative stress [54,85]. It was shown that TQ and mitochondria-targeted antioxidants, in which thymoquinone is used as the antioxidant part of the molecule, exhibit pronounced antioxidant activity in isolated mammalian mitochondria [39,86]. These experiments point to the potential antioxidant property of the TQ molecule itself, which is mediated by its high free radical scavenging activity (Figure 1). At the same time, there is some problem with the optimal neuroprotective concentration of TQ, since this quinone (as with many other antioxidants) can have both an antioxidant and a pro-oxidant effect [87]. For example, pretreatment with TQ (1.17–37.5 μM) reduced cytotoxicity in PC12 cells caused by serum/glucose deprivation [88], but 40 μM of TQ induced the death of cultured cerebellar granule neurons [89]. Oxidant/antioxidant effects of TQ depend on its concentration (Figure 2). As a quinone, TQ can be reduced by various reductases to semiquinone (one reduction) or thymohydroquinone (two reductions). Although thymohydroquinone has antioxidant properties, semiquinone can be a pro-oxidant due to its formation of ROS [90,91].

In addition, the sensitivity of different cells to the prooxidant action of TQ can vary significantly [88]. Thus, further studies of the mechanisms of antioxidant and pro-oxidant action of TQ and the development of optimal pharmacological forms of the drug are currently needed.

## 5. Conclusions

In neurodegenerative processes, the most important link in the multidimensional cascades of neuron damage is oxidative stress, which is a therapeutic target that can be affected by neuroprotectors. One of such substances is thymoquinone, which is able not only to prevent the development of oxidative stress, but also to maintain the membrane potential of mitochondria, activate the autophagy process, and lower the level of pro-inflammatory cytokines.

The results obtained on various models of neurodegenerative diseases and pathological conditions of the brain demonstrate a high neuroprotective and antioxidant potential of TQ; this property makes this substance a promising basis for the development of prototypes of therapeutic agents aimed at the treatment of a number of degenerative diseases of the central nervous system.

## Data Availability

Not applicable.

## Abbreviations

MDA	malondialdehyde
MPP+	1-methyl-4-phenylpyridinium
MPTP	1-methyl-4-phenyl 1,2,3,6 tetrahydropyridine
Nrf2	Nuclear factor erythroid 2-related factor 2
ROS	Reactive oxygen species
SOD	superoxide dismutase
TQ	thymoquinone
